# *Terminalia arjuna* Bark Powder as a Potential Immunomodulator in *Labeo rohita*: Enhanced Hematological, Adaptive, and Humoral Responses against Bacterial Pathogens and Concordant Liver Histomorphology

**DOI:** 10.3390/pathogens13040295

**Published:** 2024-03-30

**Authors:** Dharmendra Kumar Meena, Basanta Kumar Das, Amiya Kumar Sahoo, Narottam Prasad Sahu, Prem Prakash Srivastava, Simanku Borah

**Affiliations:** 1 ICAR-Central Inland Fisheries Research Institute, Barrackpore 700120, India; d.meena@icar.gov.in (D.K.M.); amiya.sahoo@icar.gov.in (A.K.S.); simanku.borah@icar.gov.in (S.B.); 2 ICAR-Central Institute of Fisheries Education, Mumbai 400061, India; npsahu@cife.edu.in (N.P.S.); ppsrivastava@cife.edu.in (P.P.S.)

**Keywords:** *Terminalia arjuna*, immunomodulation, hematology, adaptive immunity, humoral immunity, integrated biomarker response, liver histomorphology

## Abstract

This study investigated the dietary immunomodulatory effects of *Terminalia arjuna* bark powder (TABP) in *Labeo rohita*, a freshwater fish model. Four iso-nitrogenous and iso-caloric diets containing graded levels of TABP (0, 1, 10, and 15 g/kg were fed to fish for 90 days, followed by a 10 day challenge with pathogenic bacteria *Aeromonas hydrophila* and *Edwardsiella tarda*. An integrated biomarker response (IBR) approach assessed the impact of TABP on hematological, adaptive, and humoral immune parameters, along with liver histomorphology. Dietary TABP at 10 g/kg significantly enhanced (*p* < 0.05) hematological indices (hemoglobin, red blood cell count, hematocrit), specific immune parameters (lysosomal enzyme activity, phagocytosis, respiratory burst), and non-specific immune parameters (serum lysozyme, alternative complement activity), and exhibited improvements in liver architecture consistent with the enhanced immune response. Broken line regression analysis showed 11.5 g/kg to be an optimum dose. However, at 15 g/kg, a compromised trend was observed in some parameters. These findings suggest an optimal dosage range for TABP’s immunomodulatory effects. The study highlights the potential of TABP as a natural immunomodulator in fish aquaculture. The improved immune response and concomitant liver health observed in *Labeo rohita* opens avenues for further research on TABP’s applicability in animal health, using fish as a model organism. Additionally, the IBR approach proved effective in evaluating TABP’s immunomodulatory properties, paving the way for similar studies on other natural products in aquaculture.

## 1. Introduction

Aquaculture is a rapidly growing food system that provides nutrition and livelihood security to a large population. However, disease outbreaks are a major challenge to the aquaculture industry, causing significant economic losses. It has been reported that fish protein is considered to be more easily digestible and less expensive than other animal proteins [1]. Indian major carps account for 60–70% of total fish production, with *Labeo rohita* accounting for a sizable portion of that [2]. Due to its compatibility with other species and consumer preferences, the fish *L. rohita* plays an important role in six species composite aquaculture in South-East Asian countries including India [3]. Current fish production is insufficient to meet the rising demand, with global per capita fish consumption hovering around 20.3 kg [2]. The strategies for increasing fish production as a prime demand include deploying new culture technologies in reservoirs and wetlands alongside existing culture practices, which are dependent on several factors such as scientific farming, quality feed, seed, and effective disease management measures [4]. Klesius et al. [5] reported that 10–15% of the total value of fish production worldwide is lost due to different diseases. In India, for example, Andhra Pradesh shares a major chunk of 40,000 tons of major carps, worth INR 600 million per year, but the loss due to disease outbreak is estimated to be INR 40 million. The conventional approach to disease treatment is antibiotics, and the irrational use of chemicals and substances for growth promoters and immunity boosters has raised concerns about the expansion of disease-resilient strains of bacteria and the presence of noxious chemicals in fish flesh. The authors of [6] stated that herbal products including ethno-medicinal plants have emerged as a potential alternative to obnoxious chemicals and antibiotics used in aquaculture. Herbs can act as immunostimulants, activating innate defense machineries in fish and increasing the adaptive immune system. The authors of [7] reported that numerous studies have shown that herbal additives promote fish growth and protect against disease. *Terminalia arjuna*, also known as arjuna, is a plant native to India. Arjuna tree bark is used medicinally in animals for a variety of health benefits. The authors of [8] evaluated *Terminalia arjuna* for its antioxidant potential, while [9] investigated its antimicrobial activities. Meena et al. [10] concluded that *Terminalia arjuna* can be considered one of the beneficial plants to fish nutrition and health. Meena et al. [10] revealed that dietary TABP showed results in accordance with its serum biochemistry and other parameters. Secondary metabolites known as bioactive compounds mediate the health benefits. The authors of [11] studied the effects of the leaf extract of *Terminalia catappa* on water quality, blood profile, and survival of Betta species. The study revealed that immersing *T. catappa* leaf extract beyond 375 ppm is advantageous in enhancing survival, WBC, RBC, and Hg of Betta species. The authors of [12] discovered that *T. arjuna* has antioxidant, hypotensive, antiatherogenic, anti-inflammatory, anticarcinogenic, antimutagenic, and gastroprotective properties in humans.

A comparative study and detailed information for evaluating the health beneficial properties of this plant extracts on fish are deficient. As a result, the current study was designed to generate detailed information about the dietary effects of *Terminalia arjuna* bark-powder-based fish feed on hematological, immunoglobulin level, and humoral responses in *Labeo rohita*.

## 2. Material and Methods

### 2.1. Diet Preparation 

Four iso-nitrogenous (30.36% crude protein) and iso-caloric (423.76 kcal GE/100 g) experimental diets were formulated using the chemical composition of basic ingredients. The following are four experimental diets based on the graded bark powder level of *Terminalia arjuna*: control diet without bark powder (0.0%, 0 g/kg inclusion)(CT); 0.1% (1 g/kg inclusion) (T1); 1% (10 g/kg inclusion)(T2) and 1.5% (15 g/kg inclusion)(T3). The required quantitative feed ingredients were weighed and mixed homogeneously. The mixture was then cooked at 15 psi in a pressure cooker (Prestige Whistle Pressure Regulator Weight Whistle for All Inner Lid & Outer Lid Pressure Cookers, Stainless Steel, Silver, Kolkata, India), 1.5 Liter for 15 min. After the mixture had cooled, the rest of the ingredients were added to the dough, including fish oil, Butylated Hydroxy Toluene (BHT), sunflower oil, choline chloride, and vitamin and mineral mixture. The mixture was then mixed thoroughly to ensure proper blending. To obtain uniform-sized pellets, the combined ingredients were pressed with a 1 mm diameter of the pelletizer (Twin Screw Extruder Pellet Machine K K Life Science Chennai, India, 1.0 mm diameter). The pellets were collected in an aluminum tray and dried evenly in a rotating hot air oven (Meta-Lab Scientific Industries Mumbai, India) at 60 °C for 14 h. The dry pellets were collected and stored in an airtight polythene bag at 4 °C until required. The TABP was reported to have catechin, ellagic acid, gallic acid, (–)-epigallocatechin and 18-β-glycyrrhetinic acid as major bioactive compounds [8]. 

### 2.2. Feed Trial and Growth Monitoring 

The indoor feed trial was conducted for 90 days. A total of 700 fish juveniles of *L. rohita* were purchased from the local fish market (average weight, 20.3 ± 0.34 g) and reared in a 5000 L cement cistern for two weeks acclimatization at ICAR-CIFRI, Barrackpore, Kolkata, India. A 90 day study was designed and performed in triplicate, using a completely randomized design (CRD). In triplicate, 540 fish (average weight: 20.7 ± 0.34 g) were assigned to four different dietary treatments. Fish were reared in a flow-through system in a 500 L tank and were fed twice daily (9.30 am and 5.00 pm) up to satiation. Sampling was carried out every month to check the growth and health status of the fish.

### 2.3. Isolation of Pathogenic Bacterial Strains and Challenge Study 

Fish specimens exhibiting clinical signs of *L. rohita* disease were collected from an aquaculture farm in East Medinapur, West Bengal, India (Latitude 22.107897°, Longitude 87.907583°). Passive data collection revealed a reported 40% mortality within the total fish population. Individuals displaying clinical signs, such as body surface redness and hemorrhages, were selected and transported in aerated 50 L FRP tanks to the Fish Pathology Lab (ICAR-Central Inland Fisheries Research Institute), Kolkata, for etiological agent screening. Clinical and post-mortem examinations were conducted following established protocols. The animal utilization protocol for the experimental setup was approved by the Institutional Animal Ethics Committee, ICAR-CIFRI, Kolkata, India (IAEC/2020/02). Fish exhibiting clinical signs and body lesions were euthanized using clove oil anesthesia (Dabur, India) at 50 µL/L water concentration. Gut samples were aseptically collected and incubated in TSB (HiMedia, Thane, India) for 24 h at 28 °C. The overnight suspension was diluted to 10^−6^ and spread plated onto TSA (HiMedia, India) media. A single colony was then streaked onto a fresh TSA plate to obtain a pure culture.

In-door feed trial was conducted for 90 days followed by challenge study with *A. hydrophila* and *E. tarda* for 10 days. The pathogenic bacterial strains were grown for 24 h in a BOD incubator at 30 ℃ on tryptone soya broth (TSB). The cells were collected, washed thrice in sterile PBS before putting into PBS at a concentration of 1.97 × 10^8^ cells/mL, and were inoculated to the fish using the intraperitoneal method. Each fish was challenged with 100 μL of bacterial suspension, which corresponded to 10^5^ cells/mL. The relative survival percentages (%)and changes in behavioral morphology in challenged fishes were observed for up to 10 days. After the experiment, other parameters were analyzed (Table 1).

### 2.4. Calculation of Relative Percentage Survival (RPS)

The relative percentage survival provides an estimate of the potency of herbal extracts. The RPS was calculated as per the following equation [15]:

RPS = (1 − % mortality in treated fish) × 100)/% mortality in control(1)

### 2.5. Sampling and Blood and Serum Collection 

Fish were weighed to assess growth indices after the commencement of the trial (feeding). Final sampling was carried out to determine different response parameters. Three fish were randomly selected from each replicate and anesthetized with 0.015 mL /L of clove oil. Following anesthesia, blood samples were extracted from the caudal vein of each sample group using a 24 gauge needle and a 2 mL preheparinized syringe. The blood samples were then placed in a plastic eppendorf tube and stored at 4 °C for further analysis. Fish from each subgroup were also randomly selected for serum and blood analysis from each experimental set. These fish were anesthetized with 0.1 ppm MS 222 during sampling. Following anesthesia, blood samples were obtained from the caudal vein of each study group using a 24 gauge needle or 2 mL preheparinized syringe. The blood samples were then placed in a plastic eppendorf tube without anticoagulant for serum analysis. The blood samples were allowed to clot for 40 min on an ice pack in a slanting position. After clotting, the serum supernatant was collected and processed at −20 °C after centrifugation for 5 min at RCF 27,950× *g* in a table-top centrifuge.

### 2.6. Blood Parameters 

The blood samples were analyzed for CBC using a Sysmex XP-100 3 part differential fully automated hematological analyzer (Sysmex Corporation, Kobe, Japan). This instrument is a high-performance hematology analyzer that provides accurate and reliable results for a wide range of CBC parameters. The following parameters were estimated in the instrument.

Red blood cells (RBCs) (×106/µL), white blood cells (WBCs) (×103/µL), hematocrit (HCT) (%), hemoglobin (Hb) (g/dL), mean corpuscular volume (MCV) (fL), mean corpuscular hemoglobin (MCH) (pg), platelets (PLTs) (×103 nos./µL), mean corpuscular hemoglobin concentration (MCHC) (g/dL).

### 2.7. Enzyme-Linked Immunosorbent Assay (ELISA) for IgM

IgM levels were assessed using ELISA following the method of [16] with a few modifications. Serum preparation involved dilution in 50 mM carbonate–bicarbonate buffer (pH 9.5) at a dilution of 1:1000. The diluted serum was then coated onto ELISA plates and incubated at 4 °C overnight for plate coating. Subsequently, unbound antigens were removed by washing the plates three times with washing buffer (10 mM PBS, 0.05% Tween-20, supplied by Sigma-Aldrich, St. Louis, MO, USA). Wells were blocked with 200 µL of blocking solution (5% skimmed milk) for 1 h at room temperature (RT) to prevent nonspecific binding. Next, the plates were washed again with washing buffer and coated with fish immunoglobulin serum from immunized mice at a dilution of 1:1000, followed by incubation for 1.3 h at RT. Afterward, unconjugated antibodies were removed by washing the plates three times with washing buffer. The plates were then conjugated with anti-mouse IgM HRP (horseradish peroxidase, Sigma-Aldrich) in a 1:2000 dilution in 3% bovine serum albumin (BSA, Sigma-Aldrich) for 45 min at RT. Subsequent removal of unconjugated antibodies was performed by washing the plates three times with washing buffer. For the colorimetric reaction, a total of 100 µL of tetramethyl benzidine (TMB, Merck, Darmstadt, Germany)/H_2_O_2_ in distilled water (DW) at a ratio of 1:20 was added to each well, and the plates were incubated for 5–10 min to allow the reaction to occur. The reaction was then terminated by adding 100 µL of 2 N H_2_SO_4_ to each well. Finally, the absorbance of the wells was measured at 450 nm using an ELISA reader (Transasia Elisa reader, Mumbai, India) to quantify the levels of IgM.

### 2.8. Serum Humoral Responses 

#### 2.8.1. Respiratory Burst (NBT) Assay

The respiratory burst (RB) activity was carried out using the nitro blue tetrazolium (NBT) assay as mentioned in Rao [17]. In brief, the blood suspension (100 µL) was mixed with 0.1 mL of 0.2% NBT (Sigma) and incubated at 37 °C for 1 h. After incubation, 50 µL of NBT-blood suspension was mixed with 1.0 mL of N,N-dimethyl formamide (Himedia, India) and centrifuged at 3000 rpm for 5 min. The supernatant was collected into a glass cuvette, and absorbance was recorded at 540 nm in the BioTekEpoch^TM^ 2 microplate spectrophotometer (Santa Clara, CA, USA).

#### 2.8.2. Serum Bactericidal Assays

The procedure following Kajita [18] was used to determine the serum bactericidal assay, with minor modifications. Briefly, 36 h of fresh broth culture of *F. columnare* were centrifuged at 5000 rpm for 10 min. The harvested cells and serum (100 µL) were mixed with phosphate-buffered saline (PBS) and incubated for 1 h at 37 °C. The serum bacterial suspension was diluted with PBS at a ratio of 1:10, and the serum suspension culture was spread on nutrient agar plates and incubated for 36 h at 37 °C. In the control group, sterile PBS was used in place of serum. After incubation, the number of bacteria was determined by counting the colonies that grew on the nutrient agar plates.

#### 2.8.3. Serum lysozyme Activity

The serum lysozyme assay was determined according to Parry [19], with minor modifications. In brief, collected serum (10.l) samples were mixed with 1 mL of 107 cell/mL Micrococcus leteus (ATCC 44732) culture. The reaction was incubated at 37 °C for 15 min, and absorbance was measured in a BioTekEpoch^TM^ 2 microplate at 546 nm from 0 min to 5 min at a continuous interval of 30 sec. A unit of lysozyme activity was defined as the amount of sample causing a decrease in absorbance of 0.001/min, and the lysozyme was expressed as Unit/mL.

### 2.9. Serum Albumin, Globulin, and Microprotein

The serum parameters, such as total protein (PRO(U/L)), albumin (ALB (U/L)), and globulin (Glob(U/L)), were assessed employing fully automated biochemistry analyzer Transasia-Erba EM–2000. The A:G ratio was calculated based on the parameter values obtained from the automated biochemistry analyzer.

### 2.10. Histolarchitextural Changes

For the histopahological study, livers were isolated from the normal and experimental fish. Tissues were then fixed in a neutral formaline buffered solution of 10% and processed through a series of graded alcohols before they were cleared with choloroform and embedded in paraffin wax. With the assistance of a Leica RM2125RTS microtome, sections were trimmed at 5 micron thickness, stained with hematoxylin and eosin (Humason, 1972), and mounted in D.P.X. The image of prepared sections was taken at 100X, 200X, and 400X magnification under an axiostar carlzeiss microscope.

### 2.11. Integrated Biomarker Approach for Elucidating the Effect of Dietary TABP on Hematological, Adaptive Immunity, and Humoral Response Biomarkers in A Fish Model

To assess the multi-biomarker response in *L. rohita*, we calculated integrated biomarker responses (IBRs) for several biomarkers in hematological, humoral response, and adaptive immunity, and displayed corresponding star plots. We followed the proposed approach by [20,21]. The scores (S) of all biomarkers assessed in a specific treatment. The IBRs were calculated using the following formulae:

Ai = Si/2 sinβ (Si cosβ + Si + 1 sinβ)(2)

where β = Arc tan (Si + 1 sinα / Si − Si + 1 cosα) and α = 2π/*n*, Sn + 1 = S1.

In these formulas, Ai is the area that connects the two scores (S), Si and Si + 1 are the two successive clockwise scores (radius coordinates) of a specific star plot, and *n* is the total number of biomarkers used in the calculations. The IBR index for each treatment was standardized to account for different genes by computing the average value of each biomarker. This allowed us to compare the IBRs of different treatments across different parameters. The star plots showed that the IBRs for the different treatments were significantly different. This suggests that the multi-biomarker response in *L. rohita* is sensitive to different stressors. The IBRs can therefore be used as a tool to assess the stress levels in *L. rohita* and to identify the most sensitive biomarkers.

### 2.12. Statistical Analysis 

The data were initially analyzed in Microsoft Excel v.16 to identify and address any inconsistencies, errors, or missing values. Statistical analyses were subsequently conducted using IBM SPSS Statistics 20. The specific tests used were determined based on the nature of the data and the research objectives. Common tests employed in our research included measures of central tendency (mean, median) and dispersion (standard deviation, variance) for descriptive statistics. Normality testing was performed using the Shapiro–Wilk test to assess the normality of data distribution. If the data were not normally distributed, non-parametric tests were employed. Parametric tests such as one-way ANOVA, two-way ANOVA, t-tests (paired and independent), and the Pearson correlation coefficient were used. Non-parametric tests like the Kruskal–Wallis test, Mann–Whitney U test, and Spearman’s rank correlation coefficient were utilized. Integrated biomarker response analysis (IBR) was carried out to assess the cumulative effects of the studied parameters on *Labeo rohita* fish. This analysis allowed us to evaluate the combined impact of multiple stressors on the organism’s health and identify potential synergistic or antagonistic effects.

## 3. Results 

### 3.1. Growth Monitoring

The study found that *Labeo rohita* fish showed the highest growth rates and best nutrient use when fed 10 g TABP/kg of feed. This also led to the highest survival rate. TABP significantly improved weight gain, most significantly in the T2 group and least significantly in T3. The control group (CT) did not differ significantly from T1. Various growth parameters and nutrient use were affected by TABP. The highest WG % (148.41%) and SGR (1.51% per day), as well as PER (1.31), were observed in fingerlings fed with 10 g/kg TABP. Conversely, FCR showed a reverse trend, being highest at 15 g/kg TABP. Growth rates, feed conversion, and nutrient utilization were lowest at 15 g/kg TABP. Interestingly, the performance in these aspects was similar between the control (CT) and T3 groups (*p* > 0.05). While feed intake did not vary significantly among treatment groups (*p* > 0.05), protein and fat intake differed with TABP (*p* < 0.05). Survival rates were similar between CT and T1 (*p* > 0.05), but significantly different between T2 and T3 (*p* < 0.05). The hepatosomatic index (HSI) varied among treatments (*p* < 0.05), with T2 showing the highest values. Additionally, the gastrosomatic index (GaSI) and craniosomatic index (CSI) were highest in T2 (*p* < 0.05). Notably, T2 had the highest relative percent survival (RPS) against *A. hydrophila* (81.25), while T3 had the lowest against *E. tarda* (51.11). This order of RPS was: T2>T1>T3>CT, supporting the effects of TABP on fish physiology.

### 3.2. Relative Percentage Survival 

**Figure 1:**
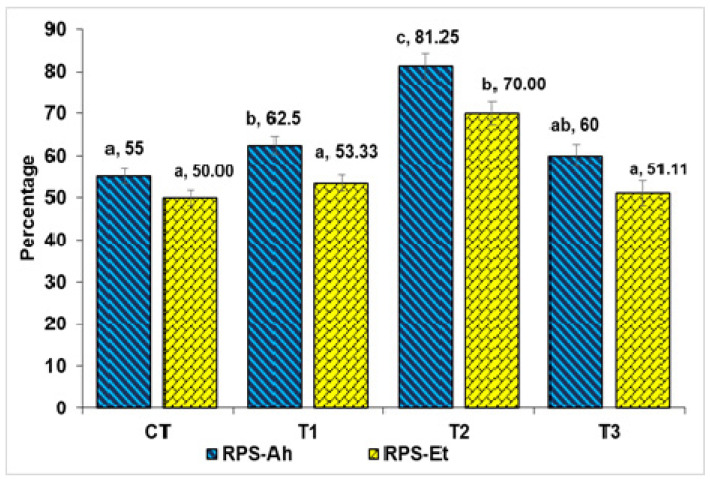
Relative percentage survival in under indoor feed trial followed by challenge study. In figure superscript represents significance at 5 % level (*p* < 0.05). The relative percentage survival in feed trial followed by two bacterial pathogens explained the variation in efficacy of TABP.

The results showed that there was a significant difference in the RPS between the treatments (*p* < 0.05). The T2 group had the highest RPS (81.25%), followed by the T1 group (75%), the T3 group (51.11%), and the CT group (45.83%). The T3 group was not statistically different from the CT or T1 groups. This suggests that the T3 group was not significantly more or less susceptible to infection with Ah or Et than the CT or T1 groups (Figure 1).

### 3.3. Humoral Responses 

**Figure 2:**
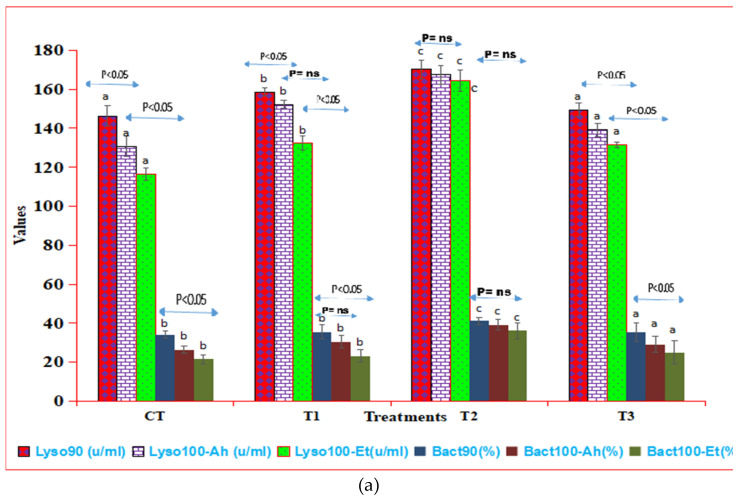

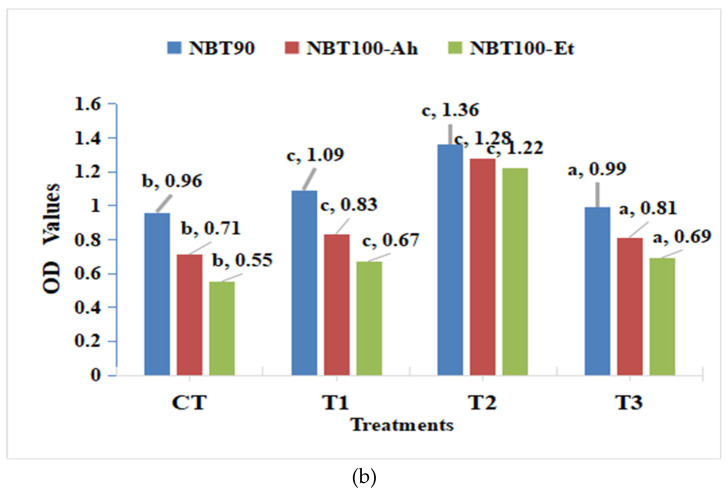
(**a**) Humoral responses in indoor feed trial and challenge study. The superscript indicates the significance between and among the treatments, whereas the arrow mark indicates the variation within the treatments before challenge and after challenge study. Here, Lys90 and Bact90, represent the values of lysozyme activity and bactericidal activity at 90 days of feeding trial. Suffix100, along with these three parameters, indicates challenge study with two pathogenic bacterial isolates, Et (*Edwardsiella tarda)* and Ah (*Aeromonas hydrophila)* at 100 days (10 days duration, 90–100 days). (**b**) Respiratory burst activity responses by NBT in indoor feed trial and challenge study. The superscript indicates the significance between and among the treatments, whereas the arrow mark indicates the variation within the treatments before challenge and after challenge study. Here, NBT 90 represents the values of respiratory burst activity at 90 days of feeding trial. Suffix100 indicates challenge study with two pathogenic bacterial isolates, Et (*Edwardsiella tarda)* and Ah (*Aeromonas hydrophila)* at 100 days (10 days duration, 90–100 days).

The feed experiment showed significant variations in immune parameters (Lys90, Bact90, NBT90) among treatment groups. Treatment 2 (T2) notably increased these parameters compared to CT and Treatment 1 (T1), while Treatment 3 (T3) decreased significantly. In the challenge study, both CT and T3 exhibited declining trends from 90 to 100 days, unlike T2, which remained stable. This pattern persisted in the challenge study involving *Aeromonas hydrophila* (Ah) and *Edwardsiella tarda* (Et), as depicted in Figure 2a,b. The consistent performance of T2 in maintaining stable values suggests its potential efficacy in enhancing resistance against these pathogens. Linear regression analysis revealed significant relationships between measured parameters and treatment duration. For NBT in T2, the regression equation was:

Y = −0.12x + 1.26 (R2 = 0.92)(3)

Similarly, for Lys90 and Bact90, the equations were:

Y = −1.87x + 162.09 (R2 = 0.99) (4)

and

Y = −0.96x + 42.09 (R2 = 1)(5)

respectively, providing predictive insights into parameter behavior under treatment. Comparing values at 90 and 100 days post-infection showed a significant decline, but no difference between CT and T3, suggesting T3 treatment did not enhance immune response beyond the control. Overall, these findings underscore Treatment 2’s effectiveness in boosting immune parameters and resistance to specific pathogens, emphasizing the need for ongoing monitoring in experimental settings.

### 3.4. Complete Blood Count (CBC)

#### 3.4.1. Hematological Parameters of *L. Rohita* under Indoor Feed Trial and Challenge Study

Table 2 shows that hematological parameters, including hematocrit (%) at 90 days (HCT90), mean corpuscular volume at 90 days (MCV90), mean corpuscular hemoglobin at 90 days (MCH90), and platelets at 90 days (PLT90), exhibited a substantially (*p* < 0.05) increasing trend in Treatment 2 (T2) when comparing feed treatments to challenge studies with *Aeromonas hydrophila* (Ah) and *Edwardsiella tarda* (Et).

However, upon infection, the trend was the same, but it was observed to be decreasing. Among the treatments, a feed trial followed by infection investigation revealed that practically all of the parameters in Control (CT) and Treatment 1 (T1) after 90 days and challenge with Et (100-Et) exhibited a substantial (*p* < 0.05) decrease in value. The values in T3 were also decreasing but exhibited no significance (*p* > 0.05). The values in T2 did not vary substantially (*p* > 0.05).

#### 3.4.2. RBC, WBC, and Hg Content in Indoor Feed Trial and Challenge Study

Red blood cell (RBC) and white blood cell (WBC) counts were significantly different between treatment groups in both the feed experiment and the challenge study. In the feed experiment, RBC90 (RBC count at 90 days) was significantly higher in Treatment 2 (T2) than in the other treatment groups (*p* < 0.05). There was no significant difference in RBC90 between CT, T1, and T3. After infection with *Aeromonas hydrophila*, RBC90 decreased significantly in all treatment groups (*p* < 0.05), but the decrease was greatest in T2. WBC90 (WBC count at 90 days) was also significantly higher in T2 than in the other treatment groups (*p* < 0.05). There was no significant difference in WBC90 between T1 and T3, but CT and T1 differed significantly (*p* < 0.05). The pattern of WBC90 after infection followed by a feed trial was similar to the pattern of RBC90, with a declining trend. However, the decline was not as steep as the decline in RBC90. 

**Figure 3:**
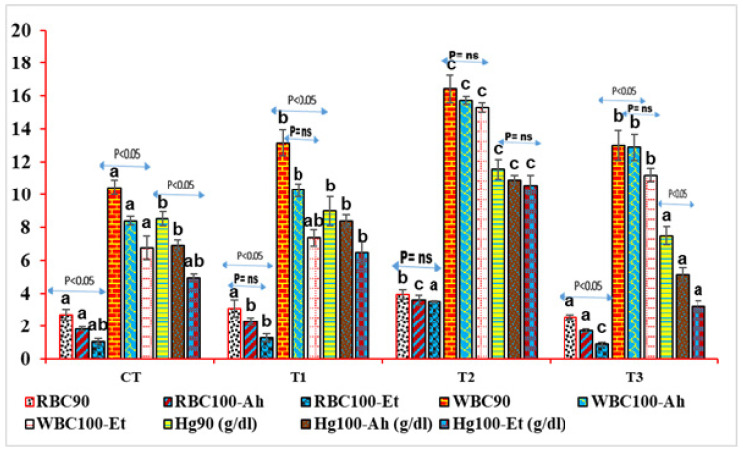
RBC, WBC, and Hg content in indoor feed trial and challenge study. Here, RBC is expressed as (×10^3^ nos./µL) and WBC as (×10^6^ nos./µL). The graph showed variation between and within the treatments. The superscript indicates the significance between and among the treatments, whereas the arrow mark indicates the variation within the treatments before challenge and after challenge study. The suffix 90 indicates time interval and 100-Ah and 100-Et indicate challenge study at 100 days with *A. hydrophila* and *E. tarda*.

The Hg levels followed the same pattern as RBC and WBC levels. In the feed experiment, Hg90 (Hg level at 90 days) was significantly higher in Treatment 2 (T2) than in the other treatment groups (*p* < 0.05). There was no significant difference in Hg90 between CT, T1, and T3. After infection with *Aeromonas hydrophila*, Hg levels decreased significantly in all treatment groups (*p* < 0.05), but the decrease was greatest in T2. Between 90 days and 100 days after infection with Ah, there were non-substantial (*p* > 0.05) variations in CT and T3, and insignificant (*p* > 0.05) variations in T2 and T1 (Figure 3).

#### 3.4.3. Protein, Albumin, Globulin, and Albumin: Globulin in Indoor Feed Trial and Challenge Study

Albumin (ALB), protein (PRO), and globulin (Glob) levels varied significantly among treatment groups in both the feed experiment and the challenge study. ALB90 was notably higher in Treatment 2 (T2) compared to other groups (*p* < 0.05), while no significant difference was observed between CT and T1. PRO90 increased significantly in T2 (*p* < 0.05) but decreased in T3 (*p* < 0.05), with significant differences among all groups (*p* < 0.05). Glob90 differed significantly between CT and T1 (*p* < 0.05) but not between T2 and T3, with T2 having the highest value followed by T1>T3>CT.

**Figure 4:**
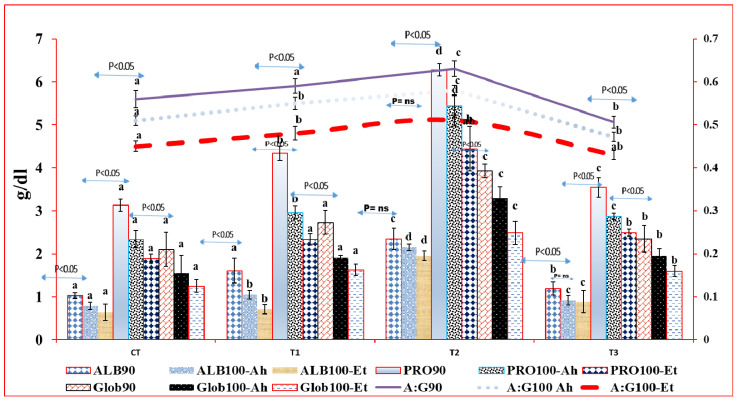
Protein, albumin, globulin, and albumin globulin ratio in indoor feed trial and challenge study. The superscript indicates the significance between and among the treatments, whereas the arrow mark indicates the variation within the treatments before challenge and after challenge study. Here, ALB90, Glob90, Pro90, and A:G90 represent the value of albumin, globulin, total protein, and albumin–globulin ratio at 90 days of feeding trial. The suffix 100Ah and 100Et indicate the value of these parameters’ consequent upon infection with *Aeromonas hydrophila* and *Edwardsiella tarda,* respectively, at 100 days.

The Albumin–globulin (A:G) ratio differed significantly among treatment groups in both the feed experiment and the challenge study. Treatment 2 (T2) showed significantly higher A:G90 compared to other treatments (*p* < 0.05). A:G90 did not differ significantly between CT and T1. Similar trends were observed for A:G100-Ah and A:G100-Et. After feeding trial and infection with Ah and Et, all groups experienced decreased parameter values. CT and T1 showed drastic decreases (*p* < 0.05) with Ah and Et, while T1 only exhibited a significant decrease (*p* < 0.05) with Et. T2 showed no significant decrease, and T3 decreased significantly (*p* < 0.05) with Et but not considerably (*p* > 0.05) with Ah (Figure 4).

### 3.5. Microprotein

**Figure 5:**
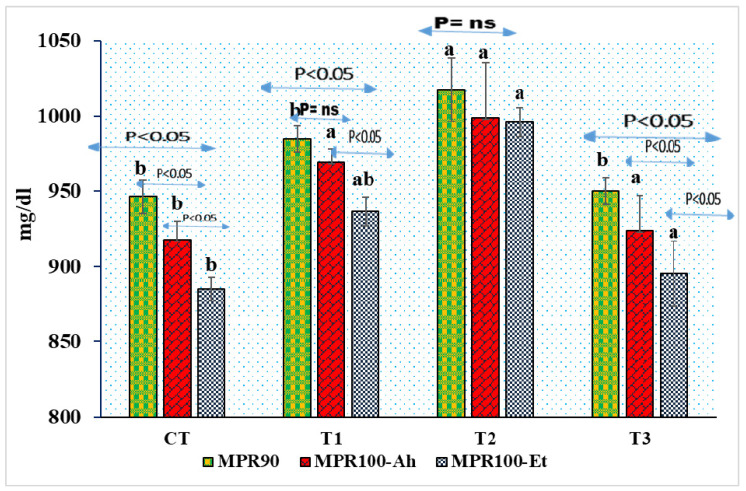
Microprotein values in indoor feed trail and challenge study. Here, superscript indicates the significance between and among the treatments, whereas the arrow mark indicates the variation within the treatments before challenge and after challenge study. The Y-axis shows the microprotein values expressed in mg/dL. The X-axis depicts the treatment groups of indoor feed trial and challenge study. The values were measured at the end of indoor feed trial (90 days) and challenge study (100 days) as shown in figure.

The microprotein levels at 90 days (MPR90) significantly increased in T2 (*p* < 0.05), with no significant difference observed between CT, T1, and T3 (*p* > 0.05). Elevated MPR90 values in T2 may be due to higher stress hormone levels. MPR100-Ah and MPR100-Et at 100 days showed no significance between T1, T2, and T3 (*p* > 0.05), indicating similar values across treatments. Infections with *A. hydrophila* and *E. tarda* decreased MPR90, MPR100-Ah, and MPR100-Et values. Positive correlation existed between MPR90 and MPR100-Et in T1 (*p* < 0.05) but not with MPR100-Ah. No correlation was found in T2 (*p* > 0.05), while in T3, a significant decrease was observed (*p* < 0.05). The linear equation for MPR in T2 is Y = −10.69x + 1025.4; R2 = 0.85, indicating a negative correlation with time. This suggests a quadratic decline in MPR100-Ah and MPR100-Et levels across all treatments, indicating decreasing MPR values over time in T2 (Figure 5).

### 3.6. Antibody Titre

**Figure 6:**
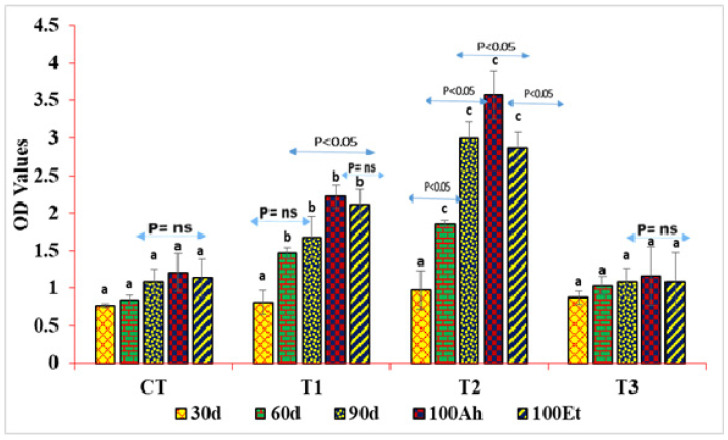
Variation in the values of immunoglobulin M (IgM) under indoor feed trial and challenge study. The superscript indicates the significance between and among the treatments, whereas the arrow mark indicates the variation within the treatments before challenge and after challenge study. The IgM values are being represented in OD values (Y-axis) measured in ELISA reader. The X-axis depicts the treatments groups of indoor feed trial and challenge study. The values were measured at 30, 60, 90, and 100 days, as shown in the figure.

The study findings revealed no significant difference in IgM levels among treatments after 30 days. However, by day 60, a notable variance emerged, with the T2 group exhibiting the highest IgM levels, followed by T1, CT, and T3 groups. Subsequently, at 100 days, IgM levels remained significantly higher in the T2 group compared to others. Interestingly, IgM levels in fish infected with *Aeromonas hydrophila* (Ah) and *Edwardsiella tarda* (Et) differed significantly across treatments, except for the control (CT) and T3 groups. Specifically, in CT, there was no significant change in Ah and Et levels. In T1, no significant difference was observed between CT-30 and CT-60, but significant differences were noted between other time points. Additionally, a significant difference was observed between 90 day and 100 day values for Ah, but not between Ah and Et (Figure 6).

### 3.7. Histoarchitectural Changes 

**Figure 7:**
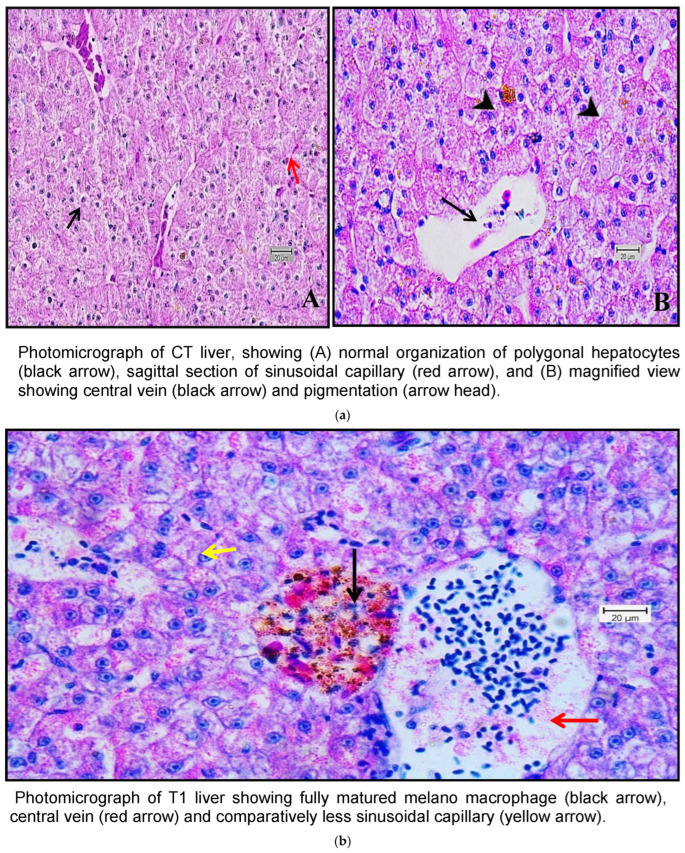

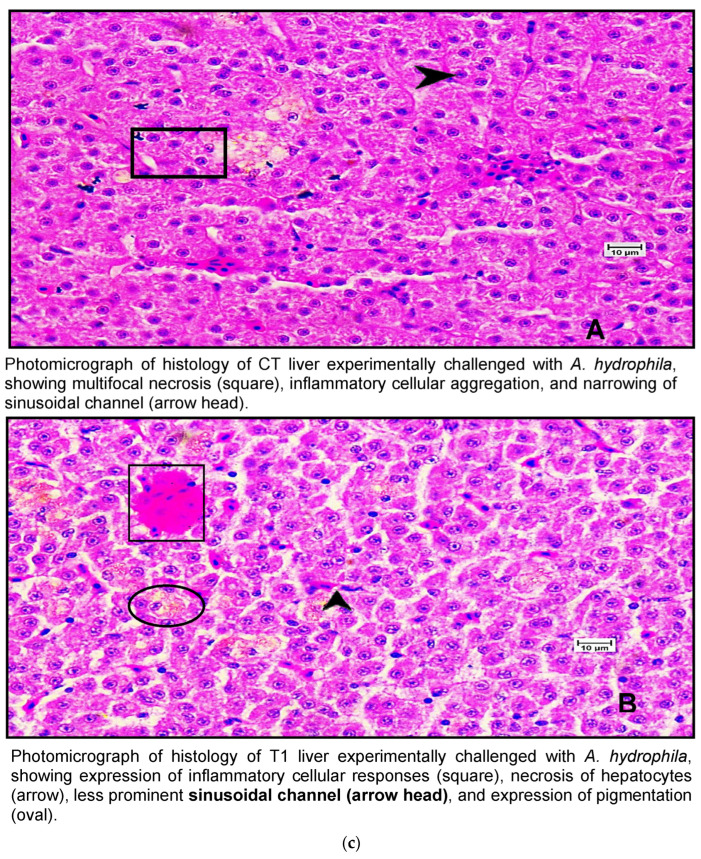

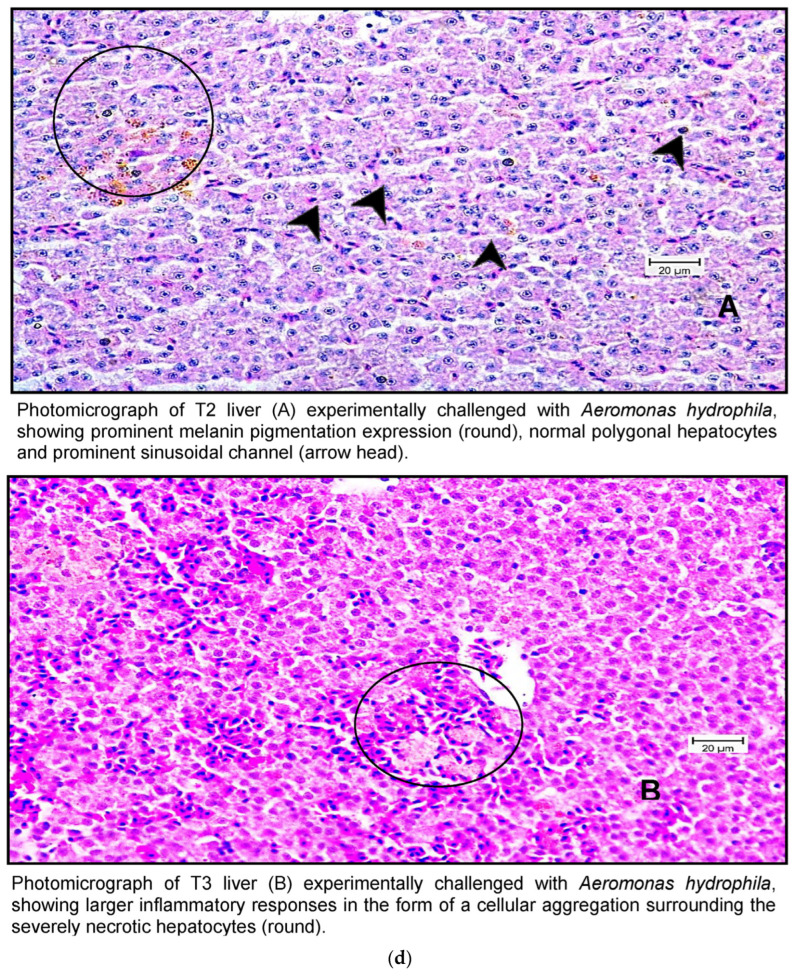

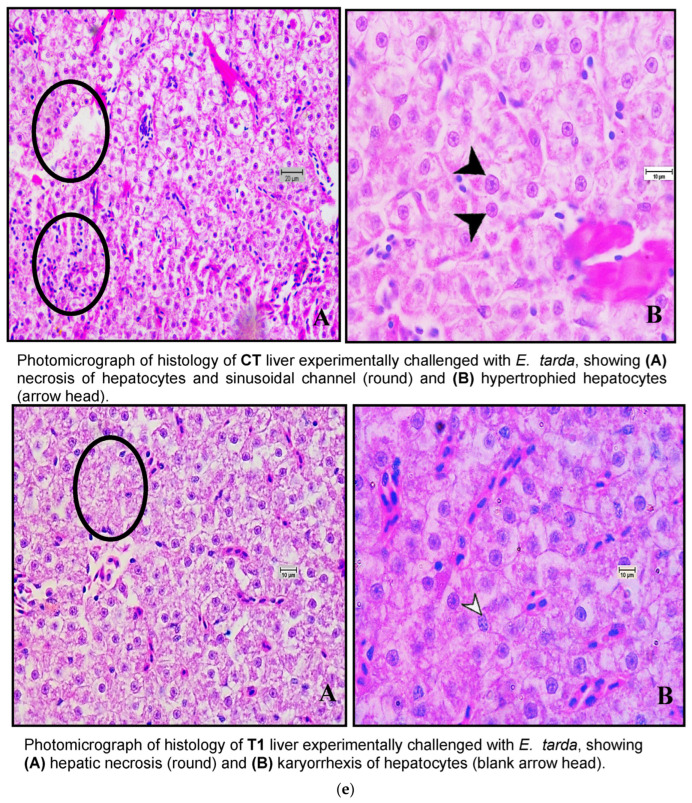

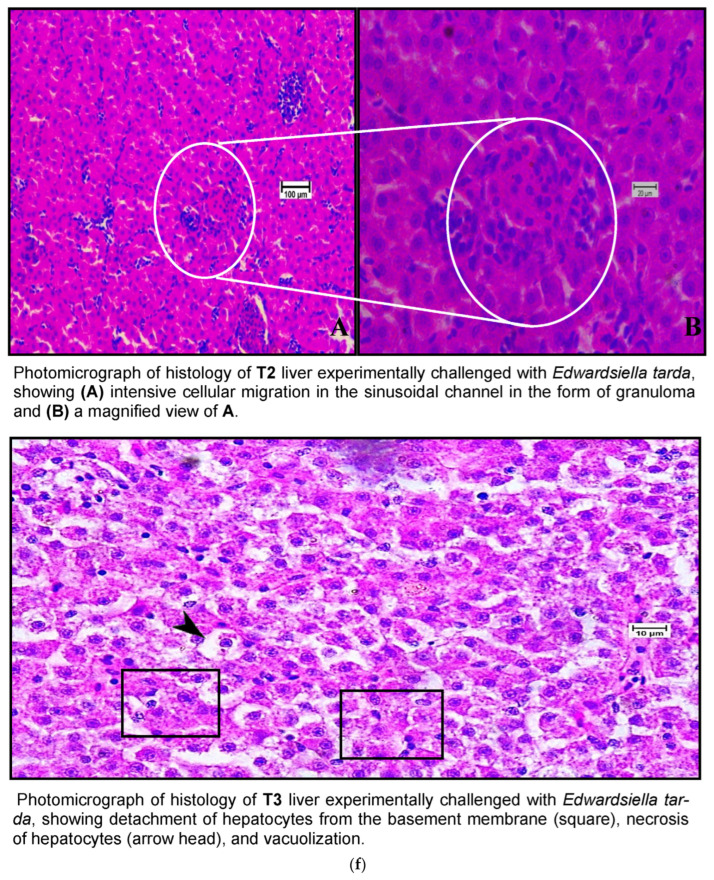
(**a**) A photomicrograph of the livers of CT and T1 groups. (**b**) A photomicrograh of the livers of T2 and T3. (**c**) A photomicrograph of the livers of CT and T1 groups challenged with *A. hydrophila*. (**d**) A photomicrograph of the livers of T2 and T3 groups challenged with *A. hydrophila*. (**e**) A photomicrograph of the livers of CT and T1 infected with *E. tarda*. (**f**) A photomicrograph of the histology of the liver of T2 and T3 infected with *E. tarda*.

Histological examination of the livers of fish fed the control diet (CT) and the high-dose TABP diet (T3) revealed significant differences in the liver pathology (Figure 7a,b). The livers of fish fed the CT diet had a normal organization of polygonal hepatocytes, normal bile duct, and central vein. In contrast, the livers of fish fed the T3 diet showed fibrosis with fat deposits and altered the normal architecture of the hepatocytes. These findings are consistent with previous studies that have shown that high doses of TABP can cause liver damage in fish. The liver is a vital organ for fish, as it plays a role in digestion, absorption, metabolism, and detoxification. Liver damage can lead to a number of problems, including impaired growth, decreased reproduction, and increased susceptibility to disease. The findings of this study suggest that high doses of TABP can cause liver damage in fish, and further research is needed to determine the safe level of exposure for fish. In addition to the histological changes, the fish fed the T3 diet also had increased levels of oxidative stress markers in the liver. Oxidative stress is a condition that occurs when there is an imbalance between the production of free radicals and the body’s ability to remove them. Free radicals are unstable molecules that can damage cells and tissues. The increased levels of oxidative stress markers in the fish fed the T3 diet suggest that TABP may be causing oxidative stress in the liver, which could contribute to the liver damage observed in this study. The findings of this study provide evidence that high doses of TABP can cause liver damage in fish. Further research is needed to determine the safe level of exposure for fish and to identify the mechanisms by which TABP causes liver damage.

### 3.8. Integrated Biomarker Approach for Hematological, Adaptive Immunity, and Humoral Response Biomarkers in a Fish Model

**Figure 8:**
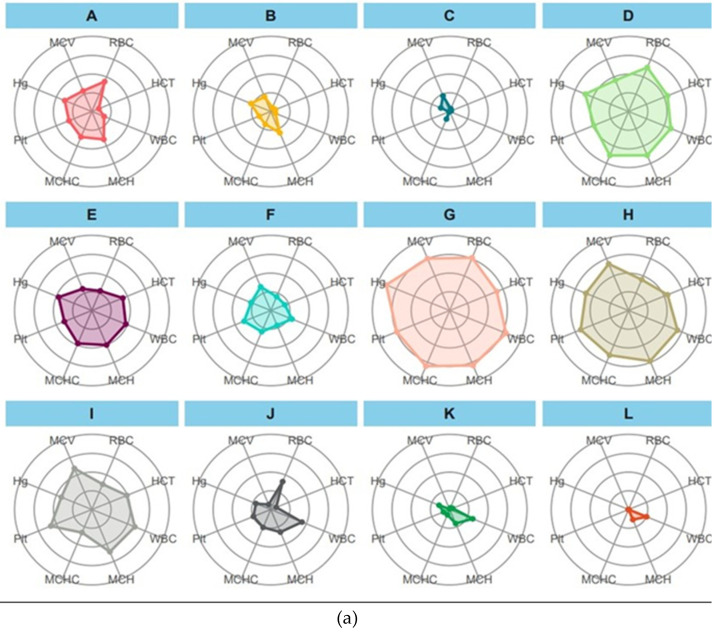

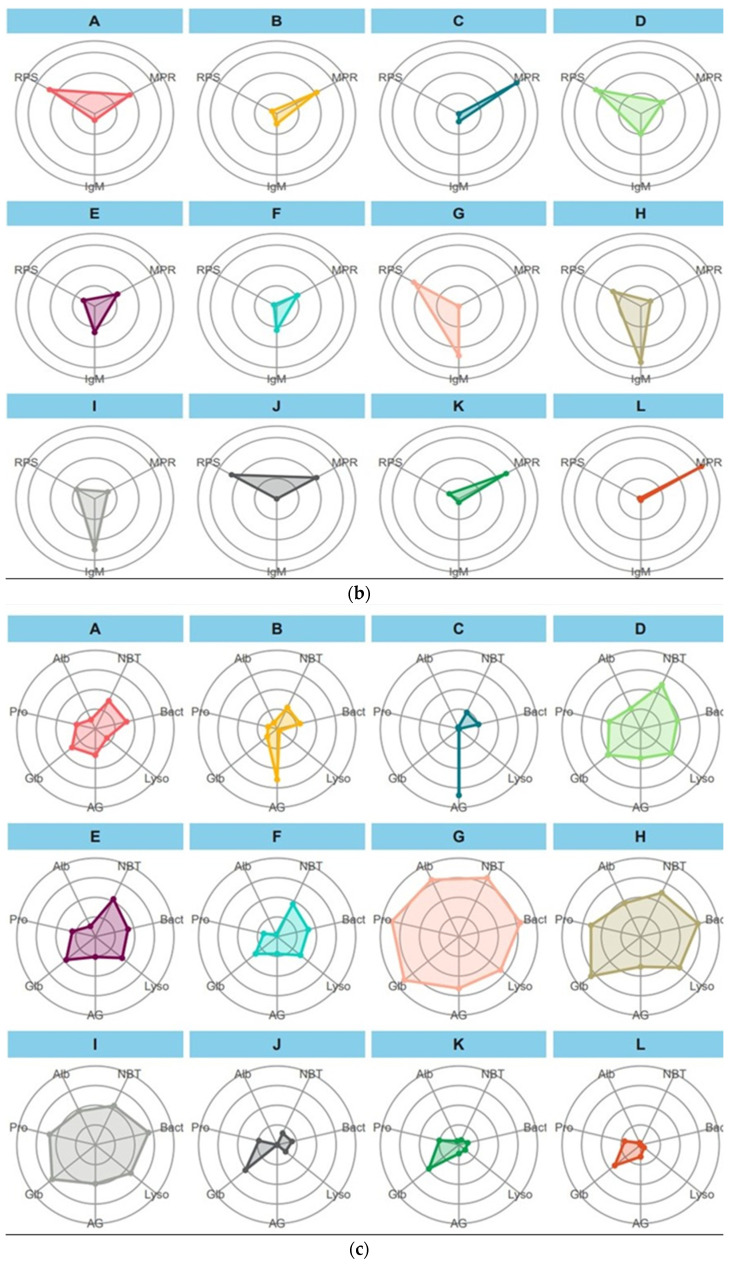
(**a**) IBR star plot of hematological markers. Here, A: CT90, B: CT100ah, C: CT100et, D: T190, E: T1 100ah, F: T1 100et, G: T2 90, H: T2 100ah, I: T2 100et, J: T3 90, K: T3 100ah, L: T3 100et, RBC—red blood cell, WBC—white blood cell, HCT—hematocrit, Hg—hemoglobin, MCV—mean corpuscular volume, MCH—mean corpuscular hemoglobin, PLT—platelets, and MCHC—mean corpuscular hemoglobin concentration. The experiment was conducted for 90 days as an indoor feed trail and then followed by a challenge study with two bacterial isolates, *Aeromonas hydrophila* and *Edwardsiella tarda*. (**b**) IBR star plot of adaptive immunity markers. Here, A: CT90, B: CT100ah, C: CT100et, D: T190, E: T1 100ah, F: T1 100et, G: T2 90, H: T2 100ah, I: T2 100et, J: T3 90, K: T3 100ah, L: T3 100et, RPS—relative percentage survival, MPR—microprotein, IgM—immunoglobulin. (**c**) IBR star plot of serum markers. Here, A: CT90, B: CT100ah, C: CT100et, D: T190, E: T1 100ah, F: T1 100et, G: T2 90, H: T2 100ah, I: T2 100et, J: T3 90, K: T3 100ah, L: T3 100et, ALB—albumin, NBT—nitroblue tetrazolium test, Lyso—lysozyme activity, Pro—protein, Glb—globulin, Bact—bactericidal activity, AG—albumin–globulin ratio.

In our study, IBR star plots depicted stress responses in fish exposed to different pollutant concentrations. Figure 8a–c illustrate IBR star plots for hematological, adaptive immunity, and humoral response biomarkers. Increased spoke length indicates heightened stress response, observed across all biomarkers in fish exposed to TABP. These changes align with known pollutant effects on fish health. Additionally, IBR star plots offer insights into relative biomarker importance and temporal dynamics of stress response. Spokes closer together suggest biomarkers acting in the same pathway, aiding in understanding stress response progression. IBR star plots serve as valuable tools for visualizing and analyzing organisms’ stress responses to environmental stressors.

### 3.9. IBR for Hematological Markers 

In this study, experimental groups are labeled with letters: A, B, and C represent the CT group, while D, E, and F stand for T1, G, H, T2 is denoted by I, and J, K, and L represent T3. This classification aids in comparing and analyzing different treatments’ effects on biomarkers. The integrated biomarker response (IBR) star plot method is used to assess biomarkers’ status visually. Each biomarker is represented by spokes extending from a central point, with length and direction indicating magnitude and change direction, respectively (Figure 8a). Examining the IBR star plot reveals distinct patterns across groups. The CT group shows a prevalence of spokes for RBC count and a larger plotted area except for WBC count and HCT. T1 displays sharper spikes and an expanded area, especially for erythrocyte parameters. Conversely, T2 exhibits the most pronounced spikes and area, suggesting a heightened biomarker response. Interpreting the plot provides insights into fish physiological status. For instance, upward-pointing WBC count spokes indicate stress, while downward-pointing spokes suggest reduced levels. When multiple biomarkers show divergent changes, their relative IBR values become crucial. Overall, group G demonstrates the healthiest hematology parameters, highlighting the IBR star plot’s utility in understanding treatment effects on fish physiology and identifying potential stressors in aquatic environments.

### 3.10. IBR for Adaptive Immunity 

The data revealed a decreasing trend in IgM and RPS in Group 1 (A, B, C), while MPR increased (Figure 7b), indicating an ineffective immune response against fish pathogens. In Group 2 (D, E, F), both MPR and RPS decreased, suggesting a similar ineffectiveness. However, a relationship between MPR and RPS was observed, implying a connection between these parameters. Group 4 (J, K, L) exhibited increasing MPR and decreasing RPS, indicating an effective but weaker immune response compared to Group 3 (G, H, I). Overall, Group 3 demonstrated the best adaptive immunity, effectively preventing serious illness. MPR90 emerged as a sensitive biomarker for stress detection in Indian major carps, declining upon infection with bacterial pathogens (Ah and Et) in all treatments, suggesting its utility as an infection biomarker.

### 3.11. IBR for Serum Parameters

The analysis of the humoral response in fish infected with IBR reveals concerning findings for Groups 1 and 4, while offering insights into immune resilience in Groups 2 and 3 (Figure 8c). In Group 1, significant drops in protein, albumin, and the albumin–globulin (A:G) ratio suggest a compromised immune system, leaving them highly susceptible to IBR infection. Continuous declines in NBT and bactericidal activity indicate a weakened ability to combat the infection, resulting in a complete breakdown of the humoral immune response. Group 2 shows some hope, with a partial humoral response indicated by a decline in albumin levels but a retained phagocytic activity suggested by NBT trends. In contrast, Group 3 exhibits the highest values across all parameters, indicating a robust and effective humoral immune response, enabling successful combat against IBR infection. Group 4, akin to Group 1, experiences compromised NBT and bactericidal activity, along with decreasing protein and globulin levels, rendering them vulnerable to IBR infection due to an ineffective humoral response.

## 4. Discussion 

### 4.1. Hematological Changes 

Fish have a two-tiered immune system consisting of innate (non-specific) and specific (humoral and cell-mediated) responses. The innate immune system is the first line of defense against pathogens, and it includes physical barriers such as mucus and skin, as well as cellular components such as phagocytes and natural killer cells. The specific immune system is activated when the innate immune system is overwhelmed, and it includes antibodies and cell-mediated responses. There is growing evidence that herbs can have a positive impact on the immune response of fish [22]. For example, a study by [23] found that the long-term feeding of herbs to fish resulted in an increase in serum total protein, haemato-biochemical, and humoral responses. Similarly, [24] found that the addition of the herbs *Curcuma longa, Mangifera indica*, and *Allium sativum* to the diet of fish enhanced their hematological parameters. The mechanisms by which herbs exert their immunostimulatory effects are not fully understood, but it is thought that they may work by increasing the production of immune cells, activating the innate immune system, and/or stimulating the production of antibodies.

The study investigated the effects of dietary *Terminalia arjuna* bark powder (TABP) on the hematological parameters and immune response of *Labeo rohita* juveniles. The results showed that TABP administration significantly increased the WBC, RBC, Hg, HCT, MCV, MCH, MCHC, albumin, globulin, and protein levels in the fish. The NBT activity and bactericidal activity also increased significantly in the TABP-treated fish. Contrastingly, ref. [25] investigated that ethanol bark extract negatively affected the hematological parameters when an overdose of the extract was given to *Heteropneustes fossilis*. Immunostimulants can boost nonspecific immunity in the host by increasing phagocyte count and NBT to combat pathogenic microbes [26]. Following infection with bacterial pathogens, NBT activity reduced non-significantly, when compared with the 90 day feed trial. These findings suggest that TABP has immunostimulatory effects in *L. rohita*. The increase in WBC levels is indicative of an enhanced innate immune response, while the increase in NBT activity and bactericidal activity suggests that TABP may also have a role in the specific immune response. The increase in serum protein and globulin levels may be due to the increased production of antibodies in the fish. These antibodies are important for fighting against infection. The results of the present study suggest that TABP can be used as a natural immunostimulant to improve the hematological parameters and immune response of *L. rohita*. This could have important implications for the aquaculture industry, as it could help to reduce the incidence of disease in fish populations.

The findings of the present study are consistent with previous research that has shown that TABP has immunostimulatory effects in other animals. For example, a study by [27] found that dietary *Stachys lavandulifolia* extract could improve blood indices in *Cyprinus carpio*. The erythrocyte count increased after TABP administration, which could be attributed to TABP’s immunostimulatory effects. Previous research [28] (found that rohu fed garlic-containing diets for 60 days had significantly higher erythrocyte counts.

Ref. [29] discovered that when channel catfish were fed a glucan-based diet, the number of erythrocytes was considerably greater (*p* < 0.05) than when they were fed a control diet. The presence of leucocytes is known to help trigger the immune system in rohu following immunostimulation application [24,28,30]. The antimicrobial properties of traditional herbs, such as garlic, are supported by an increase in total white blood cells, and other cell types, upon feeding of herbs [31,32]. The low level of hemoglobin in treatments could reflect physiological alternations to a lower demand of oxygen and the substrate for metabolism in an oxygen-stressed environment thereby leads to fluctuations in the pH of the blood [33]. The decrease in hemoglobin levels in the T3 group may be due to a number of factors, including hemopoiesis exhaustion under hypoxic conditions, breakdown of blood cells due to temperature and salt stress, or infection with bacteria such as *Aeromonas hydrophila* or *Edwardsiella tarda*. The results of the present study suggest that dietary TABP can improve the hematological parameters of *L. rohita* juveniles, but that the optimal dose may need to be lower than 15 g TABP/kg feed to avoid negative effects on hemoglobin levels.

### 4.2. Humoral Responses

Immunostimulants have been reported to augment humoral responses by enhancing the efficiency and number of cell types for lysozyme synthesis per cell in fish [34]. The number and type of stressors to which the fish are subjected have a significant impact on changes in lysozyme activity [35]. After ingesting immunostimulants, several fish species had higher lysozyme levels. For example, [36] found that feeding 0.5 percent garlic to hybrid tilapia (*Oreochromis niloticus x Oreochromis aureus*) for 2 or 4 weeks resulted in a similar increase in lysozyme activity. Several studies have also discovered that phagocytic cells from various fish species have increased bactericidal activity [28,32]. The NBT test is a measure of an oxygen-reliant protective machinery in vertebrate phagocytic cells that yields responsive oxygen intermediates with great antimicrobial responses [37]. The authors of [38] discovered that increased NBT is linked to increased phagocyte bacterial pathogen killing activity, and thus improved immunity.

In the present study, the effects of dietary *Terminalia arjuna* bark powder (TABP) on the lysozyme activity and bactericidal activity of *Labeo rohita* juveniles were investigated. The results showed that TABP administration significantly increased the lysozyme activity and bactericidal activity of the fish. The increase in lysozyme activity was likely due to the enhanced efficiency and number of cell types for lysozyme synthesis per cell [39]. The increase in bactericidal activity was likely due to the increased production of reactive oxygen species by phagocytic cells. The authors of [40] discovered that when juvenile gilthead seabream was subjected to repetitive stresses, their respiratory burst activity decreased. According to [41], *L. rohita* reared at higher temperatures also had lower respiratory burst activity. Further, lower doses of herbal stimulants resulted in better survival against pathogenic challenges in fish, according to [28,42]. When *Penaeus monodon* were fed an Artemia-rich herbal diet containing a blend of five plants, this resulted in the improvement of their health status [43]. The results of the present study suggest that TABP can be used as a natural immunostimulant to improve the humoral and cellular immune responses of *L. rohita* juveniles. This could have important implications for the aquaculture industry, as it could help to reduce the incidence of disease in fish populations. However, it is important to note that the optimal dose and duration of TABP administration need to be further investigated. Additionally, the mechanisms by which TABP exerts its immunostimulatory effects need to be elucidated. Overall, the results of the present study provide valuable insights into the potential use of TABP as a natural immunostimulant in aquaculture. Further research is needed to confirm the findings and to develop optimal protocols for the use of TABP in aquaculture.

### 4.3. Antibody Titre (IgM) and MicroProteins (MPRs) 

Immunoglobulins (Igs), or antibodies, are a key component of the specific immune system in fish. They are heterodimeric glycoproteins that belong to the broad Ig superfamily. In bony fishes, three classes of heavy chains have been identified: IgM, IgD, and IgT/Z. However, not all fish species express all three classes of Igs [44]. The levels of Igs in fish vary among species and with age. For example, IgM levels are typically higher in larger fish than in smaller fish. This is because IgM is the first line of defense against pathogens, and it is produced in greater quantities as fish grow and mature. The levels of Igs can also be affected by environmental factors, such as stress and temperature [45]. For example, fish that are stressed may have lower levels of Igs, as their immune system is not functioning as effectively. The levels of Igs can be used as an indicator of the health of fish. For example, a decrease in IgM levels may indicate that a fish is infected with a pathogen. Herbal-based immunomodulators are a new approach to improving the health of fish [46]. These immunomodulators can help to increase the levels of Igs in fish, which can help to protect them from infection [47].

The oral method is promising for immunostimulation in Indian major carps. Immunomodulators in feed activate non-specific immune systems, while vaccines induce prolonged protection. This study investigated oral immunomodulation’s effects on IgM levels in Indian major carps, finding IgM useful as a biomarker for stress and infection. The T2 group had significantly higher IgM levels, indicating heightened stress or infection, while the T3 group showed lower levels. An increase in IgM levels in the T2 group after 60 days suggested enhanced immune response, while a decline in the T3 group implied suppressed response. However, fluctuations in IgM levels over time imply its limited reliability.

### 4.4. Integrated Biomarker Approach 

The integrated biomarker approach is a multi-parameter approach that uses a combination of hematological, adaptive immunity, and humoral response biomarkers to assess the health status of fish. This approach is more comprehensive than using a single biomarker, as it can provide a more complete picture of the fish’s immune response [48]. The hematological biomarkers that are typically measured in this approach include white blood cell (WBC) count, hematocrit, and hemoglobin. These biomarkers can provide information about the fish’s overall immune status, as well as the specific type of immune response that is being mounted [49]. The adaptive immunity biomarkers that are typically measured in this approach include antibody titers and cytokine levels [50]. Antibody titers can provide information about the fish’s ability to mount an immune response against a specific pathogen, while cytokine levels can provide information about the activation of different immune cells. The humoral response biomarkers that are typically measured in this approach include lysozyme activity and complement levels. Lysozyme is an enzyme that can break down the cell walls of bacteria, while complement is a system of proteins that can help to kill bacteria and other pathogens. The integrated biomarker approach has been used to study the health status of fish in a variety of settings, including aquaculture, environmental monitoring, and disease research. This approach has been shown to be a valuable tool for assessing the immune status of fish and for identifying potential health problems.

The findings from the study regarding the adaptive immunity of fish affected by IBR align with earlier research. In a prior investigation [51], it was observed that the levels of IgM and RPS decreased in bacterial-infected fish, whereas the levels of MPR increased. This indicates that IgM may not be a dependable indicator of the fish’s capability to generate an effective immune response against bacterial pathogens. Conversely, MPR emerges as a more reliable measure of the fish’s immune health in response to bacterial pathogens. The difference in RPS between the treatments may be due to the different stressors that were applied to the fish. The T2 group was exposed to a moderate stressor, which may have made them more resistant to infection. The T3 group was exposed to a severe stressor, which may have made them more susceptible to infection. The results of this study suggest that the RPS can be used as a biomarker for detecting stress and infection with bacterial pathogens in Indian major carps. The T2 group had the highest RPS, suggesting that they were more resistant to infection with Ah or Et. The T3 group had the lowest RPS, suggesting that they were more susceptible to infection with Ah or Et.

The results of the study also suggest that there is a relationship between MPR and RPS. This is consistent with the findings of [52], who found that MPR and RPS were positively correlated in fish infected with pathogens. This suggests that the two parameters are likely to be affected by the same factors, such as the severity of the infection and the fish’s individual immune response. Overall, the results of the study provide further evidence that biomarkers can have a negative impact on the adaptive immunity of fish. However, the severity of the impact appears to vary depending on the fish’s individual immune [53] response. Further studies are needed to better understand the mechanisms by which IBR affects the adaptive immunity of fish, and to develop effective strategies for preventing and treating the disease. The results of the study on the humoral responses of fish infected with bacterial isolates are consistent with previous studies. For example, ref. [54] found that the levels of protein, albumin, and A:G decreased in fish infected with bacterial isolates, while the levels of NBT and bactericidal activity showed a constant decrease. This suggests that bacterial isolates can have a negative impact on the humoral immunity of fish [55].

Bacterial isolate infection reduces humoral immune parameters in rainbow trout; IgG, IgM, and IgT levels decrease significantly in infected fish. Vaccination can mitigate this impact, suggesting a link between infection severity and immune response. Fish with severe infection show the most significant decrease in immune parameters. Severity of bacterial isolate infection negatively affects fish’s humoral immune response. This study is the first to illustrate TABP’s immunomodulatory effects in fish, with ongoing research exploring its effects in other animals [56]. The immunomodulatory effects (antibody titre) of herbal product/ medicinal plants have been reported in other fish species i.e., tilapia [57], carps [58], *Channa punctatus* [59], shrimps [60], and cat fish [61].

## 5. Conclusions

In summary, the study highlights *Terminalia arjuna* bark powder (TABP) as a promising immunomodulator in *Labeo rohita* fish. TABP supplementation, notably at 10 g/kg over 90 days, significantly improved various immune parameters, enhancing both specific and non-specific immune responses crucial for pathogen defense. These findings suggest sustained effects of TABP, indicating its potential to boost fish immune function. Conducted under controlled conditions with a large sample size, the study’s robustness lends credibility to its implications for diverse fish populations and potential application in aquaculture practices in vivo safe level determination by the same author in previous research [62]. TABP emerges as a compelling candidate for further exploration in enhancing fish health through natural immunomodulation.

## Figures and Tables

**Table 1:** Feed formulation and chemical composition of experimental diets.

Ingredients (g/kg)	CT	T1	T2	T3
Fish meal	5.00	5.00	5.00	5.00
Groundnut oilcake	16.00	16.00	16.00	16.00
Soybean meal	28.00	28.00	28.00	28.00
Mustard oilcake	13.50	13.70	14.00	14.20
De-oiled rice bran	14.50	14.50	14.50	14.50
Wheat flour	14.95	14.25	13.45	12.75
Fish oil	3.00	3.00	3.00	3.00
Sunflower oil	3.00	3.00	3.00	3.00
* Vitamin mineral mix	2.00	2.00	2.00	2.00
Choline chloride	0.02	0.02	0.02	0.02
Phytase	0.01	0.01	0.01	0.01
Butylated hydroxytoluene	0.02	0.02	0.02	0.02
^1^ TABP	0.00	0.50	1.00	1.50
Total	100.00	100.00	100.00	100.00
^1^ Chemical composition of the diets (% dry matter basis)				
Dry matter	92.37 ± 1.15	93.27 + 1.64	94.12 + 1.89	94.55 + 1.96
Crude protein	30.24 ± 0.24	30.78 ± 0.10	30.45 ± 0.16	30.89 ± 0.27
Ether extract	6.92 ± 0.08	6.85 ± 0.05	6.78 ± 0.09	7.02 ± 0.12
Ash	9.98 ± 0.07	10.14 ± 0.17	9.87 ± 0.13	10.54 ± 0.09
Crude fiber	8.34 ± 0.02	8.96 ± 0.15	9.44 ± 0.07	10.09 ± 0.05
Nitrogen free extract	44.52 ± 0.23	43.27 ± 0.16	43.46 ± 0.27	41.46 ± 0.14
^#^ Gross energy (kcal/100 g)	433.32	434.51	433.56	433.32
** Digestible energy (kcal/100 g)	361.32	357.85	356.66	352.58
P: E ratio (mg protein/ kcal DE)	83.69	86.01	85.38	87.61

^1^ Data expressed as mean (*n* = 3); ^1^ TABP—*T. arjuna* bark powder. * Composition of vitamin mineral mix (Premix Plus) (quantity/kg feed): vitamin B_2_—40 mg; vitamin E—15 mg; vitamin A (retinol)—33 mg; vitamin D_3_ (cholecalciferol)—0.55 mg; vitamin K—20 mg; vitamin B_6_—20 mg; vitamin B_12_—0.12 mcg; calcium pantothenate—50 mg; nicotinamide—200 mg; choline chloride - 3000 mg; Mn—540 mg; I—20 mg; Fe—150 mg; Zn—100 mg; Cu—40 mg; Co—9 mg; L-lysine—200 mg; DL-methionine—200 mg; selenium—2.5 mg. ^#^ The gross energy (GE) contents of crude protein, crude fat, and total carbohydrate were computed using gross calorie values of 23.6, 39.5, and 17.2 kJ/g, respectively [13] ** Digestible energy (kcal/100g) = Protein (%) × 4 + Lipid (%) × 9 + Carbohydrate (%) × 4 [14].

**Table 2:** Showing hematological parameters of *L. rohita* under indoor feed trial and challenge study.

Trt/Parameters	CT	T1	T2	T3	* *p*-Value
HCT90 (%)	34.2 ± 0.76 ^a^	37.67 ± 6.23 ^ab^	43.74 ± 4.53 ^c^	34.83 ± 0.26 ^a^	0.003
HCT100-Ah (%)	31.66 ± 0.37 ^a^	35.15 ± 5.27 ^ab^	41.81 ± 2.7 ^c^	33.25 ± 0.15 ^a^	0.003
HCT100-Et (%)	29.91 ± 0.34 ^a^	30.41 ± 0.38 ^a^	40.86 ± 2.71 ^b^	32.54 ± 0.15 ^c^	0.004
***p*-value**	0.003	0.005	0.08	0.007	
MCV90(fL)	123.66 ± 5.65 ^a^	126.88 ± 7.91 ^ab^	136.08 ± 3.06 ^b^	117.45 ± 6.11 ^a^	0.006
MCV100-Ah(fL)	120.32 ± 5.65 ^a^	123.1 ± 6.26 ^a^	134.97 ± 3.06 ^b^	115.67 ± 6.11 ^a^	0.005
MCV100-Et(fL)	116.69 ± 5.65 ^a^	1118.25 ± 5.51 ^ab^	132.78 ± 2.26 ^c^	115.48 ± 6.11 ^a^	0.005
***p*-value**	0.004	0.005	0.08	0.007	
MCH90(pg)	37.92 ± 0.58 ^a^	38.96 ± 0.58 ^a^	44.3 ± 2.15 ^b^	36.67 ± 2.31 ^a^	0.005
MCH100-Ah(pg)	33.37 ± 1.15 ^a^	35.26 ± 1.81 ^a^	43.27 ± 1.03 ^b^	34.64 ± 2 ^a^	0.005
MCH100-Et(pg)	26.34 ± 5.57 ^a^	30.88 ± 3.04 ^a^	41.81 ± 1.8 ^b^	33.72 ± 0.63 ^a^	0.005
***p*-value**	0.003	0.003	0.06	0.006	
MCHC90(g/dL)	36.21 ± 1.47 ^a^	37.97 ± 1.51 ^a^	42.4 ± 2.52 ^b^	34.67 ± 1.15 ^a^	0.005
MCHC100-Ah(g/dL)	33.62 ± 1.47 ^a^	35.38 ± 1.51 ^a^	41.14 ± 2.65 ^b^	32.08 ± 1.15 ^a^	0.005
MCHC100-Et(g/dL)	28.54 ± 1.47 ^ab^	31.3 ± 1.51 ^bc^	40.73 ± 0.59 ^c^	31 ± 1.15 ^a^	0.005
***p*-value**	0.003	0.003	0.06	0.006	
PLT90(×10^3^ nos./µL)	37.69 ± 1.44 ^ab^	40.73 ± 1.88 ^b^	51.19 ± 2.73 ^c^	35.2 ± 2.72 ^a^	0.005
PLT100-Ah × 10^3^ nos./µL)	32.66 ± 1.9 ^a^	38.6 ± 2.19 ^b^	49.92 ± 0.71 ^c^	30.19 ± 1.24 ^a^	0.005
PLT100-Et × 10^3^ nos./µL)	25.72 ± 2.26 ^a^	34.19 ± 5.39 ^b^	47.89 ± 3.47 ^c^	27.41 ± 3.84 ^a^	0.005
** *p*-value	0.003	0.003	0.06	0.006	

Values are expressed as the mean ± SE, *n* = 3; mean values in the same column with different superscripts differ significantly (*p* < 0.05) when compared to each other. Different superscripts in the same row showed significance (* *p* < 0.05) among the treatments at 0.05 level. Different superscripts in a column showed significance (** *p* < 0.05) of interaction within the treatment at 0.05 level and indicated by *p*-values. The suffix 90 indicates time interval and 100-Ah and 100-Et indicate challenge study at 100 days with *A. hydrophila* and *E. tarda,* respectively.

## Data Availability

The data will be made available based on the request.
